# Prolonged, brain-wide expression of nuclear-localized GCaMP3 for functional circuit mapping

**DOI:** 10.3389/fncir.2014.00138

**Published:** 2014-11-26

**Authors:** Christina K. Kim, Andrew Miri, Louis C. Leung, Andre Berndt, Philippe Mourrain, David W. Tank, Rebecca D. Burdine

**Affiliations:** ^1^Princeton Neuroscience Institute, Princeton UniversityPrinceton, NJ, USA; ^2^Department of Molecular Biology, Princeton UniversityPrinceton, NJ, USA; ^3^Department of Psychiatry and Behavioral Sciences, Stanford UniversityStanford, CA, USA; ^4^Center for Sleep Sciences, Stanford UniversityStanford, CA, USA; ^5^Department of Bioengineering, Stanford UniversityStanford, CA, USA

**Keywords:** transgenic zebrafish, genetically encoded calcium indicators, *in vivo* calcium imaging, brain-wide expression, nuclear calcium signals

## Abstract

Larval zebrafish offer the potential for large-scale optical imaging of neural activity throughout the central nervous system; however, several barriers challenge their utility. First, ~panneuronal probe expression has to date only been demonstrated at early larval stages up to 7 days post-fertilization (dpf), precluding imaging at later time points when circuits are more mature. Second, nuclear exclusion of genetically-encoded calcium indicators (GECIs) limits the resolution of functional fluorescence signals collected during imaging. Here, we report the creation of transgenic zebrafish strains exhibiting robust, nuclearly targeted expression of GCaMP3 across the brain up to at least 14 dpf utilizing a previously described optimized Gal4-UAS system. We confirmed both nuclear targeting and functionality of the modified probe *in vitro* and measured its kinetics in response to action potentials (APs). We then demonstrated *in vivo* functionality of nuclear-localized GCaMP3 in transgenic zebrafish strains by identifying eye position-sensitive fluorescence fluctuations in caudal hindbrain neurons during spontaneous eye movements. Our methodological approach will facilitate studies of larval zebrafish circuitry by both improving resolution of functional Ca^2+^ signals and by allowing brain-wide expression of improved GECIs, or potentially any probe, further into development.

## Introduction

Larval zebrafish are a valuable model organism for studying neuronal circuits and their development (Kinkhabwala et al., 2011; Miri et al., 2011a; Hocking et al., 2012; Warp et al., 2012). A primary reason for this is their small size and transparency, which makes them uniquely amenable to optical techniques. In contrast to other vertebrate systems, deep brain structures can be imaged non-invasively in awake, behaving larval zebrafish (Appelbaum et al., 2010; Naumann et al., 2010), and recent advancements have enabled whole-brain functional imaging at cellular resolution (Ahrens et al., 2012, 2013; Freeman et al., 2014; Portugues et al., 2014; Vladimirov et al., 2014). Stimulation of optogenetic probes can also be used throughout the nervous system to perturb neuronal activity during behavior (Arrenberg et al., 2009).

However, a number of factors constrain the utility of the larval zebrafish model. Currently there are no means for prolonged, ~panneuronal expression of molecular probes later into development. The HuC promoter (Park et al., 2000), which is commonly used to drive ~panneuronal probe expression across the brain, is most active at early larval stages (Sato et al., 2006), limiting experiments with this promoter beyond ~6 days post-fertilization (dpf). While work has been performed in older ~24 dpf zebrafish expressing a HuC-driven genetically-encoded calcium indicator (GECI), dense expression in this strain was restricted to certain regions, precluding whole-brain imaging (Jetti et al., 2014). Additionally, GECIs such as GCaMP3 are excluded from the nuclei of neurons (Tian et al., 2009). As the somata of larval zebrafish neurons are occupied almost entirely by the nucleus, signals are confined to the cytoplasmic membrane, which reduces the cross-sectional area from which functional fluorescence signals can be measured. As an alternative to using GECIs, synthetic calcium indicators can be bolus-loaded into larvae to allow robust nuclear and cytoplasmic indicator expression. However, these dye injections are invasive, lack specificity, and limit indicator expression to a time scale of hours (Stosiek et al., 2003).

Distel et al. (2009) developed a method using a modified Gal4-UAS system to potentially solve the first of these problems. They generated an optimized version of Gal4VP16 (KalTA4) that minimizes VP16-associated toxicity (Baron et al., 1997) and the silencing of reporter expression (Goll et al., 2009), and developed a UAS-reporter construct that enables self-sustaining KalTA4 expression (Kaloop). In the Kaloop construct, the desired reporter gene is followed by coding sequences for a viral T2A peptide (Provost et al., 2007) and KalTA4. After translation of the construct, an unstable peptide bond in T2A breaks and produces stochiometric expression of both the reporter gene and KalTA4. Thus even after the original promoter driving KalTA4 expression shuts off, expression of the reporter gene is maintained.

Here we build upon the Distel et al.’s (2009) results, reporting the creation of transgenic zebrafish strains exhibiting dense, neuronal, nuclear-localized expression of GCaMP3 across the brain up to at least 14 dpf. We generated a KalTA4 driver strain using the ~panneuronal HuC promoter, and Kaloop reporter strains expressing a modified version of GCaMP3 with a nuclear localization signal (nls; Bengtson et al., 2010; Schrödel et al., 2013; Freeman et al., 2014; Vladimirov et al., 2014). Neuronal probe expression in larvae was verified using cellular resolution two-photon laser scanning microscopy (2PLSM). Functionality of GCaMP3 in these larvae was confirmed by identifying putative oculomotor neural integrator cells whose fluorescence fluctuations were correlated with spontaneous eye movements. Coupling our brain-wide KalTA4 strain with other Kaloop reporter strains should enable brain-wide expression of any probe throughout later stages of development.

## Materials and methods

### Creation of vectors

***4xK + GC3***: The 4xKaloop plasmid (Distel et al., 2009) was a gift from Reinhard Köster (Institute of Developmental Genetics), and CMV-GCaMP3 was a gift from Loren Looger (Janelia Farms). The entire 4xKaloop plasmid excluding the KGFP cassette was amplified using the following primers:

- **3′** ACCGGTATGAATTCCGAATTCGTGTGGAGGAGCTCAAAGTGAGG **5′**
- **5′** ATGAATTCGCATGCACTAGTGAGGGAAGAGGAAGTCTGC **3′**

The GCaMP3 ORF was amplified from CMV-GCaMP3 using the following primers:

- **3′** ATGAATTCGCATGCCTTCGCTGTCATCATTTGTACAAAC **5′**
- **5′** ATGAATTCACCGGTCGCCACCATGATGGGTTCTCA **3′**

These two products were digested with *Age*I/*Sph*I and ligated together to generate 4xK + GC3.

***4xK* + *NnlsGC3* and *4xK* + *CnlsGC3***: To generate 4xK + NnlsGC3, the entire 4xK + GC3 plasmid was amplified while appending an nls (Kwan et al., 2007) to the N terminus of its GCaMP3 coding sequence using the following primers:

- **5′** ATGAATTCACCGGTATGGCTCCAAAGAAGAAGCGTAAGGTAATGGGTTCTCATCATCATCATCATC **3′**
- **3′** ATGAATTCACCGGTCATGGTGGCGACCGGTATG **5′**

The product was then digested with *Age*I and re-ligated. To generate 4xK + CnlsGC3, the 4xK + GC3 plasmid was amplified while appending an nls to the C terminus of its GCaMP3 coding sequence with the following primers:

- **5′** ATGAATTCGCATGCATGGCTCCAAAGAAGAAGCGTAAGGTAACTAGTGAGGGAAGAGGAAGTC **3′**
- **3′** ATGAATTCACCGGTCATGGTGGCGACCGGTATG **5′**

The product was then digested with *Sph*I and re-ligated.

***1xK + NnlsGC3***: To generate a version of 4xK + NnlsGC3 with only 1 UAS instead of 4, primers annealing to the last UAS in 4xK + NnlsGC3 were created. 4xK+NnlsGC3 was amplified with the following primers:

- **5′** ATGAATTCCCTAGGCGGAGTACTGTCCTCCGAGTCTAGAGG **3′**
- **3′** ATGAATTCCCTAGGCCTGTTATCCCTAAGCTCCAG **5′**

The amplified product was digested with *Avr*II and re-ligated to generate 1xK+NnlsGC3.

***CMV vectors***: CMV-K + GC3 was constructed by PCR amplifying the GCaMP3-T2A-KalTA4-GI-pA segment of 4xK + GC3 and subcloning it into the MCS of pcDNA3.1. CMV-K + NnlsGC3 and CMV-K + CnlsGC3 were similarly constructed from 4xK+NnlsGC3 and 4xK + CnlsGC3, respectively, with nls sequences included in the amplified segments in both cases.

***HuC:KalTA4***: pTolmini plasmid as described by Distel et al. (2009) was regenerated from 4xKaloop via a *Not*I digestion followed by re-ligation of the vector backbone. A KalTA4-GI-pA fragment was PCR amplified from 4xKaloop and subcloned into the MCS of pTolmini. The 9-kb HuC promoter was then PCR amplified from HuC-cameleon (a generous gift from Higashijima et al. (2003)) and subcloned into the pTolmini MCS upstream of KalTA4-GI-pA.

### Cell culture experiments

HEK293 cells were cultured in 6-well plates as previously described (Thomas and Smart, 2005). Confluent HEK293 cells were transfected with CMV constructs using Lipofectamine 2000 (Invitrogen, Carlsbad, CA, USA). Forty eight hours post-transfection, the wells were washed with HEPES Ringer Solution (in mM: 135 NaCl, 5.4 KCl, 1.8 CaCl_2_-Dihydrate, 5.0 HEPES salt, 1.0 MgCl_2_6H_2_O), and cells were imaged using 2PLSM on a custom-modified Olympus BX51 using a 40×/0.8 numerical aperture (NA) water-immersion objective (Olympus, Center Valley, PA, USA). 256 × 256 pixel fluorescence time series were collected in ScanImage v3.7 (Pologruto et al., 2003). Four frames sampled at ~2 Hz (2 ms per line) were collected every 10 s. After the seventh iteration of four collected frames, ionomycin (Invitrogen) was added to the well to a concentration of 20 μM.

For each construct, we analyzed the fluorescence time series of cells sampled from different wells using custom scripts written in Matlab v7.9 (Mathworks, Natick, MA, USA). Regions of interest (ROIs) were manually defined for individual cells. Fluorescence averaged over ROI pixels was normalized by its mean baseline value during the 28 frames collected prior to the addition of ionomycin to calculate fractional fluorescence changes (ΔF/F). Brightfield and epifluorescence images were captured on the Olympus BX51 using a charged-coupled device (CCD) camera (CoolSNAP CF; Roper Scientific, Trenton, NJ, USA) and RS IMAGE v1.9.2 (Roper Scientific).

### Neuronal culture experiments

Dissociated rat hippocampal neurons were cultured and transfected using a previously described protocol (Berndt et al., 2014). After 6–10 days, hippocampal neurons were transfected with a PCR-amplified portion of 1xK + NnlsGC3 containing only the nls sequence through the ORF of GCaMP3. Approximately 1 week post-transfection, the neurons were imaged using 40×/0.8 NA objective (Olympus) with a Spectra X Light excitation source (475 nm; Lumencor, Beaverton, OR, USA), and a Rolera XR camera (QImaging, Surrey, Canada) coupled to an Olympus BX61 WI microscope. Imaging was performed at 10–20 Hz using Q8apture Pro7 (Qimaging). To provide electrical stimulation to the neurons, an IsoFlex stimulus isolator (A.M.P.I., Jerusalem, Israel) was used to deliver 5–10 ms, 400–500 pA current pulses through a bipolar matrix electrode (parallel configuration with 305 μm spacing; FHC, Inc., Bowdoin, ME, USA). Intracellular recordings were performed with glass patch pipettes (4–6 MΩ; Sutter Instruments, Novato, CA, USA) pulled from a horizontal puller (P-2000; Sutter Instruments). The internal pipette solution contained (in mM): 150 KGluconate, 2 MgCl, 10 HEPES salt, 10 EGTA. Cells were kept in an extracellular tyrode (157 μM) solution containing (in mM): 135 NaCl, 4 KCl, 2 MgCl, 2 CuCl, 30 Glucose, 12 HEPES salt. A MultiClamp700B amplifier and pClamp10.3 (Molecular Devices, Sunnyvale, CA, USA) was used to make recordings.

To analyze functional fluorescence changes, ROIs were manually defined for each cell. Fluorescence averaged over the ROI pixels was normalized by its mean baseline value (fluorescence at the start of trial). Slow changes in fluorescence (bleaching artifacts) were removed by subtracting either a linear or exponential fit to the baseline fluorescence excluding GCaMP transients. During some recordings, additional 10 AP stimuli were delivered near the end of the protocol. These late-measurements were excluded from the calculation of the mean peak ΔF/F response, as they exhibited a smaller change in fluorescence due to a rundown in signal caused by bleaching. They were not excluded from calculation of *t*_1/2_on and *t*_1/2_off, as the kinetics measured from these later time points were not statistically different from those measured earlier in the recordings (Student’s 2-tailed *t*-test, *p* > 0.05). All data were analyzed in Matlab v2013a.

### Transgenic zebrafish lines

Zebrafish were reared in the Burdine Laboratory Fish Facility at Princeton University, and in the Mourrain Laboratory Fish Facility at Stanford University. All procedures involving animals were performed in accordance with Princeton University’s and Stanford University’s Institutional Animal Care and Use Committee. Heterozygous Tg[HuC:Gal4VP16] transgenic fish (Kimura et al., 2008) were obtained from Shin-ichi Higashijima (Okazaki Institute for Integrative Bioscience). Tg[HuC:GCaMP3] fish were provided by Drew Robson and Jen Li (unpublished strains, Harvard University). Tg[UAS:Kaede] (Scott et al., 2007) were provided by Herwig Baier (Max Planck Institute of Neurobiology).

To generate novel transgenic strains, we injected 25 ng/μl of plasmid DNA, 200 ng/μl Tol2 transposase mRNA transcribed from PCS2FA-transposase (Kwan et al., 2007) using the mMESSAGE mMACHINE SP6 Kit (Ambion, Austin, TX, USA), and 0.02% phenol red into 1–2 cell stage embryos. 4xK+NnlsGC3 and 1xK+NnlsGC3 were injected into embryos from Tg[HuC:Gal4VP16] × *nacre*^−/−^ (Lister et al., 1999) crosses, and HuC:KalTA4 was injected into embryos from Tg[UAS:Kaede] × *nacre*^−/−^ crosses. Injected embryos were imaged under epifluorescence (Leica MZ16FA, 2.0× lens) at 2 dpf, and were raised to adulthood if they demonstrated large numbers (roughly >100) of green fluorescent cells in the central nervous system (CNS). Upon reaching sexual maturity, injected fish were mated with wildtype fish to identify transgenic founders whose F1 progeny exhibited fluorescent cells.

### Imaging of transgenic larvae using 2PLSM

To characterize expression patterns of GCaMP3, transgenic larvae were imaged at 2 and 14 dpf under epifluorescence (Leica) using constant image capture settings. Larvae were also imaged at 6 and 10 dpf under 2PLSM on a custom-built microscope utilizing 920 nm excitation light (Cameleon Ultra II laser, Coherent, East Hanover, NJ, USA) as previously described (Miri et al., 2011b). Briefly, larvae were embedded in a thin layer of 1.7% low melting point Seaplaque agarose (Lonza, Basel, Switzerland), dorsal side up. A rectangular agarose block containing the fish was cut out and mounted on the surface of a Sylgard platform positioned within a water-filled chamber. The beam was scanned through a 60×/0.9 NA objective (Olympus) that projected laterally through a latex diaphragm into the chamber. GCaMP3 expression in the caudal hindbrain was assessed using averages of 10, 256 × 256 pixel sagittal plane fluorescence images collected in ScanImage. The same laser power was used across 6 and 10 dpf images for a given strain.

Functional GCaMP3 fluorescence signals in 6–8 dpf transgenic larvae were assessed during spontaneous eye movements, or saccades (Beck et al., 2004). Larvae were prepared as described above, except the agarose was cut free from around the eyes. We synchronously measured eye position and cellular fluorescence fluctuations in hindbrain neurons on our custom-built microscope as previously described (Miri et al., 2011b). Fluorescence image time series consisting of 500, 256 × 256 pixel images were collected in ScanImage at 2 ms per line.

### Analysis of 2PLSM fluorescence time series

All analysis of functional imaging data was performed using custom-written scripts in Matlab. To identify cells whose neuronal activity-induced fluorescence fluctuations were correlated with eye movement, we used a method we have previously described (Miri et al., 2011b). Briefly, eye position, thresholded eye velocity, and the full frame fluorescence time series were used as the variables in a linear regression model fit to each pixel’s fluorescence time series. Prior to solving the regression equation, eye position and thresholded eye velocity were convolved with an estimate of the Ca^2+^ impulse response function (CIRF). The CIRF models the change in intracellular Ca^2+^ levels in response to an action potential (AP) as an instantaneous rise and exponential decay. This convolution transforms a behavioral variable into an estimate of the Ca^2+^ fluctuation to which a firing rate encoding that variable should give rise.

In larval zebrafish, electrically recorded position cells exhibit a burst of firing at the onset of an ipsiversive saccade, a slowly decaying firing rate during the fixation that follows, and then quickly stop firing following a contraversive saccade (Miri et al., 2011b). These firing patterns translate into a steep rise in Ca^2+^-sensitive fluorescence following ipsiversive saccades, and a slower exponential-like decay in fluorescence following contraversive saccades. To measure the CIRF time constant (*τ*), we calculated the saccade triggered average fluorescence (STAF) during both ipsiversive and contraversive saccades for each cell. We only analyzed cells whose STAFs exhibited the characteristic sharp increase in fluorescence following ipsiversive saccades and a slow decay in fluorescence following contraversive saccades. An exponential fit to the contraversive STAF during the first 2 s following the saccade was used to estimate the CIRF *τ*. We identified a mean CIRF *τ* of 0.57 s (*n* = 21 cells), and used this value for all subsequent analysis.

## Results

### Nuclear localization of GCaMP3 in cell culture

**Figure 1:**
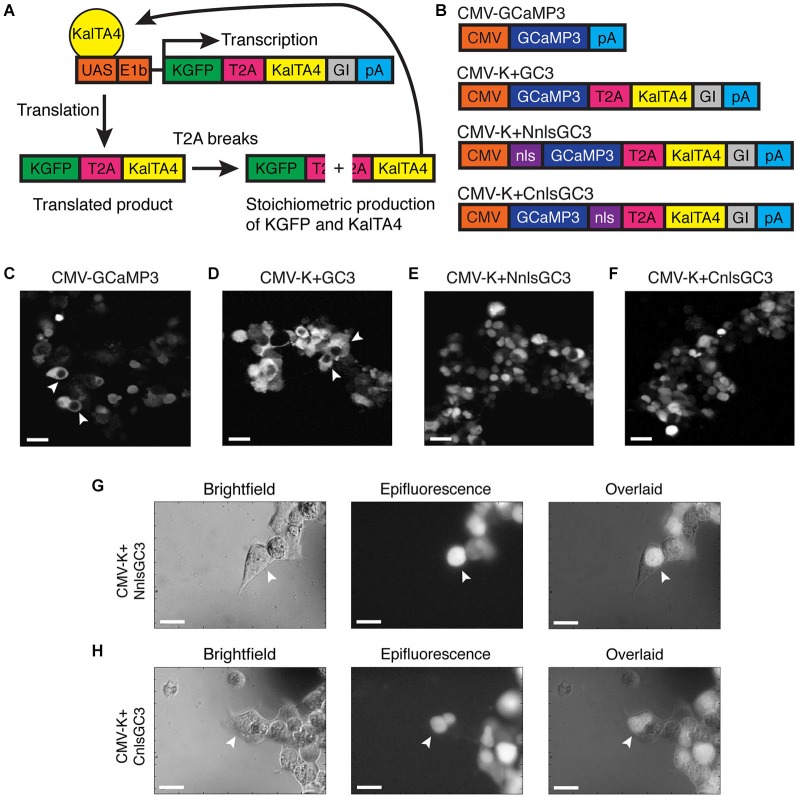
**Nuclear expression of CMV-K + NnlsGC3 and CMV-K + CnlsGC3 in HEK293 cells. (A)** Schematic of KalTA4 positive feedback mechanism in Kaloop constructs expressing KGFP. **(B)** Summary of constructs used to transfect HEK293 cells. **(C–F)** 2PLSM fluorescence images of CMV-GCaMP3, CMV-K + GC3, CMV-K + NnlsGC3, and CMV-K + CnlsGC3 expressed in HEK293 cells. CMV-GCaMP3 and CMV-K + GC3 exhibit nuclear exclusion of GCaMP3, while CMV-K + NnlsGC3 and CMV-K + CnlsGC3 exhibit nuclear inclusion. White arrowheads in **(C)** and **(D)** point to cells with clear nuclear exclusion of GCaMP3. Scale bars represent 31 μm. **(G, H)** Brightfield and epifluorescence images of HEK293 cells transfected with CMV-K + NnlsGC3 and CMV-K + CnlsGC3. White arrowheads point to cells with GCaMP3 fluorescence confined to the nucleus. Scale bars represent 20 μm.

We addressed the limited time window of ~panneuronal promoter activity and the nuclear exclusion of GECIs by utilizing a modified Gal4-UAS system developed by Distel et al. (2009), and by adding an nls to the GECI, GCaMP3. Distel et al.’s 4xKaloop construct allows constitutive labeling of cells with KGFP (GFP with a 5′ Kozak sequence) in the presence of the modified Gal4 transcriptional activator, KalTA4. KalTA4 has also been shown to reduce the mosaicism observed with the Gal4-UAS system. 4xKaloop incorporates 4 UAS repeats and the minimal E1b promoter, followed by coding sequences for KGFP, a T2A peptide, KalTA4, and a rabbit beta-globin intron fragment (GI) downstream of KalTA4 (Figure 1A). The unstable T2A peptide in the translated product breaks to produce stoichiometric amounts of both KGFP and KalTA4. KalTA4 can then feedback to bind to the UAS and continue to induce transcription of the insert, even after the promoter initially driving KalTA4 is no longer active.

We replaced KGFP in 4xKaloop with GCaMP3 (4xK + GC3), and then added an nls to either the N or C terminus of GCaMP3 (4xK + NnlsGC3 and 4xK + CnlsGC3). Prior to generating transgenic zebrafish strains, we assessed whether GCaMP3 in our constructs was properly localized and functional *in vitro*. We inserted the segment of our three new constructs spanning from GCaMP3 through the polyA cassette into the MCS of the pcDNA3.1 plasmid, generating constructs with our GCaMP3 variants under the control of the CMV promoter (CMV-K + GC3, CMV-K + NnlsGC3, and CMV-K + CnlsGC3; Figure 1B). We included the T2A and KalTA4 sequences in these constructs to ensure that their presence does not stymie nuclear localization or indicator functionality.

We then transfected our constructs and a control construct, CMV-GCaMP3, into HEK293 cells. Cells transfected with CMV-GCaMP3 or CMV-K+GC3 exhibited clear nuclear exclusion of GCaMP3 (Figures 1C,D), whereas cells expressing GCaMP3 constructs with an nls exhibited fluorescence in the nuclei of cells (Figures 1E,F). Nuclear localization of GCaMP3 was confirmed by overlapping brightfield images with epifluorescence images of cells transfected with CMV-K+NnlsGC3 or CMV-K+CnlsGC3 (Figures 1G,H). This demonstrates that the nls targets GCaMP3 to the nucleus when placed on either the N or C terminus of GCaMP3.

### Screening functionality of nuclear-localized GCaMP3 variants in cell culture

We then assessed whether the functionality of GCaMP3 was affected by the addition of T2A-KalTA4-GI or the nls. We added 20 μM ionomycin to transfected HEK293 cells and measured changes in ΔF/F using 2PLSM. Ionomycin has been demonstrated to increase the intracellular concentration of Ca^2+^ in mammalian cells (Liu and Hermann, 1978), and should elicit increased fluorescence emission from GCaMP3.

**Figure 2:**
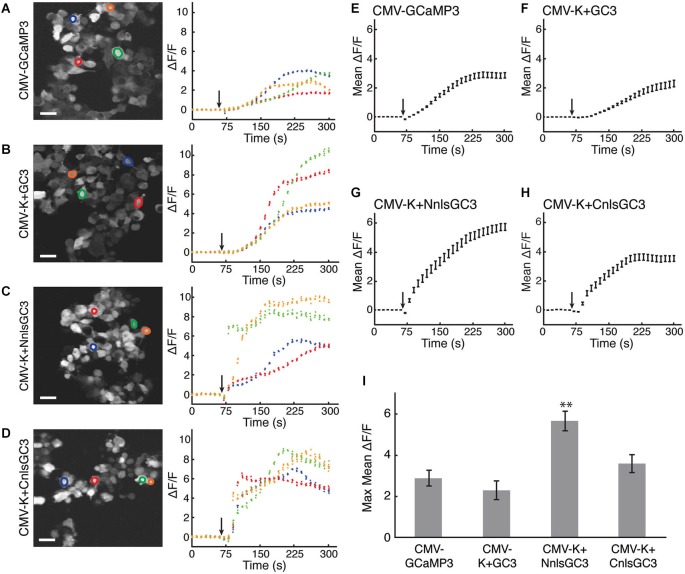
**HEK293 cells transfected with CMV-K + NnlsGC3 exhibit the largest fluorescence increase in response to ionomycin. (A–D)** 2PLSM fluorescence images (left panels) and fluorescence time series (right panels) for four example cells for each construct. Black arrows indicate when 20 μM ionomycin was added to the well. Scale bars represent 31 μm. **(E–H)** 2PLSM fluorescence time series averaged across 30 cells for each construct. Black arrows indicate when ionomycin was added to the well. Data points represent mean ± SEM. For clarity, the average of each iteration of four frames was calculated across the cells. **(I)** The maximum fluorescence increase in response to ionomycin was averaged across 30 cells for each construct. Error bars represent ± SEM. The asterisk indicates significant difference relative to all other measurements (*p* < 0.01, 2-tailed Student’s *T*-test).

Ionomycin caused increased fluorescence emission relative to baseline for all constructs. The fluorescence time series in response to ionomycin are shown for four example cells from each CMV construct in Figures 2A–D. Mean ΔF/F traces were calculated using 30 cells transfected with each construct (Figures 2E–H). The maximum of the mean ΔF/F of CMV-K + NnlsGC3 (5.68 ± 0.48, mean ± standard error of the mean, SEM) was significantly greater than that of CMV-GCaMP3 (2.88 ± 0.39), CMV-K + GC3 (2.29 ± 0.46), and CMV-K + CnlsGC3 (3.61 ± 0.44; Figure 2I; 2-tailed Student’s *t*-test, *p* < 0.01). The maximum of the mean ΔF/F of CMV-K + CnlsGC3 was also significantly greater than that of CMV-K + GC3 (Figure 2I; 2-tailed Student’s *t*-test, *p* < 0.05). This suggests that the nuclear localization and tandem expression of KalTA4 did not alter the functionality of GCaMP3 in terms of the amplitude of the fluorescence response. Based on these results, we chose 4xK + NnlsGC3 rather than 4xK + CnlsGC3 for subsequent testing and transgenesis. As high expression levels of GCaMP3 have been associated with toxicity *in vivo* (Tian et al., 2009) and may slow down fluctuations in intracellular free Ca^2+^ concentration, we also generated a version of our construct with only 1 UAS instead of 4, 1xK + NnlsGC3.

### NnlsGC3 can reliably report action potentials in cultured neurons

**Figure 3:**
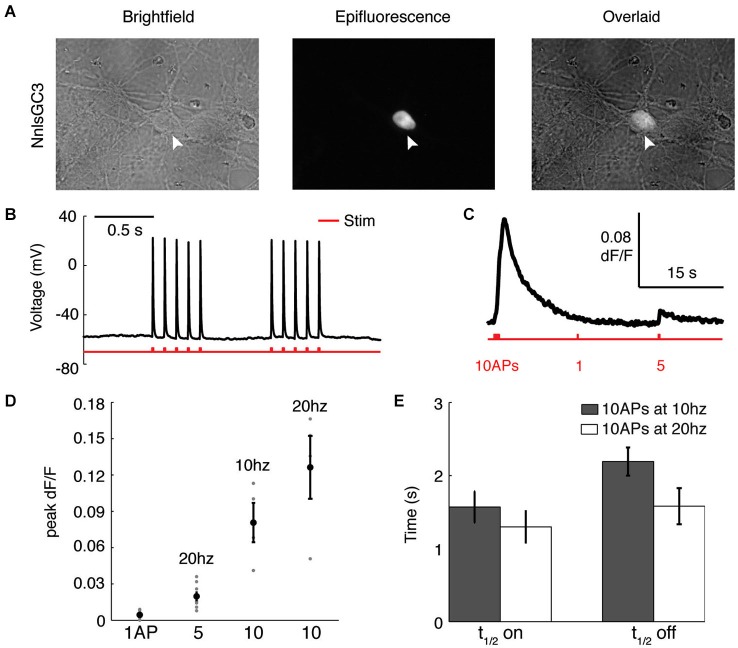
**NnlsGC3 reliably reports action potentials in cultured neurons. (A)** Brightfield and epifluorescence images of dissociated rat hippocampal neurons transfected with NnlsGC3. White arrowheads point to a cell in which clear nuclear inclusion of GCaMP3 is observed. **(B)** Example intracellular whole-cell recording of a cultured neuron in response to 10 ms, 500 pA electrical field stimulation pulses. **(C)** Fluorescence time series of a cell’s response to 10 APs at 10 Hz, 1 AP, and then 5 APs at 20 Hz (500 pA 5 ms pulse). **(D)** Mean peak change in fluorescence in response to 1 AP, 5 APs at 20 Hz, 10 APs at 10 Hz, and 10 APs at 20 Hz. Error bars represent ± SEM. **(E)** Mean *t*_1/2_on and *t*_1/2_off in response to 10 APs at 10 and 20 Hz.

To characterize the ability of our probe to report APs, we used dissociated rat hippocampal neurons transfected with NnlsGC3. As in HEK293, we observed localization of GCaMP3 to the nucleus of neurons (Figure 3A). During intracellular whole-cell recordings (Figure 3B), we first identified an extracellular field stimulation protocol that reliably induced single APs, finding that 5–10 ms pulses of 400–500 pA were sufficient. Recorded neurons were then imaged as they were driven to fire different patterns of APs (Figure 3C). We found that NnlsGC3 can reliably detect a 5 AP 20 Hz stimulus (0.020 ± 0.0030 mean peak ΔF/F; *n* = 9 trials pooled from seven cells), and 10 AP stimuli at 10 Hz (0.081 ± 0.016 mean peak ΔF/F; *n* = 4 trials pooled from three cells) and 20 Hz (0.13 ± 0.026 mean peak ΔF/F; *n* = 4 trials pooled from four cells; summarized in Figure 3D). The kinetics of NnlsGC3 fluorescence we observed (Figure 3E) were slightly slower than those reported for GCaMP3 (Tian et al., 2009; Walker et al., 2013), though expression level differences could be the cause. In response to a 10 AP stimulus at 10 Hz, we observed a mean *t*_1/2_on of 1.57 ± 0.21 s and a mean *t*_1/2_off of 2.19 ± 0.19 s (*n* = 9 trials pooled from three cells). We recorded a mean *t*_1/2_on of 1.30 ± 0.21 s and a mean *t*_1/2_off of 1.58 ± 0.25 s in response to a 10 AP stimulus at 20 Hz (*n* = 9 trials pooled from seven cells). These results suggest that NnlsGC3 can reliably report APs.

### Prolonged, but mosaic, expression of GCaMP3 with Tg[HuC:Gal4VP16]

Because the 4xKaloop construct upon which our constructs are based is flanked by Tol2 transposon fragments (Kawakami, 2005), we were able to use Tol2-mediated trangenesis to generate zebrafish reporter strains Tg[4xK + NnlsGC3] and Tg[1xK + NnlsGC3]. When coupled to a transient ~panneuronal Gal4 driver line, such as Tg[HuC:Gal4VP16], these Kaloop reporter strains should allow prolonged, expression of GCaMP3 in almost all neurons later into development. We assessed persistence of fluorescence across the CNS between 2 and 14 dpf, and variegation of labeling at 6 and 10 dpf by crossing our transgenic strains and the Tg[HuC:Gal4VP16] driver line, which exhibits ~panneuronal expression of Gal4VP16 (Köster and Fraser, 2001). For comparison, we also evaluated the transgenic strain Tg[HuC:GCaMP3], which does not incorporate the KalTA4-Kaloop reporter system and exhibits ~panneuronal expression of the GCaMP3 at early larval stages.

**Figure 4:**
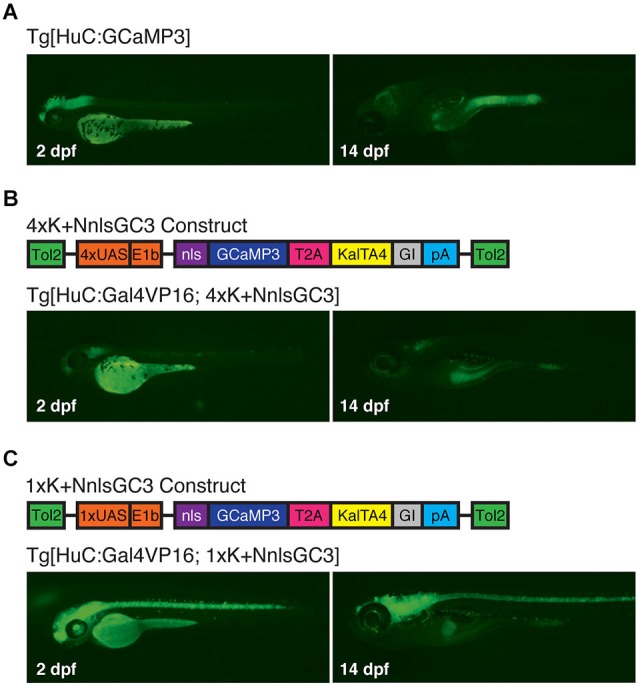
**Tg[HuC:Gal4VP16; 1xK + NnlsGC3] larvae exhibit robust expression of GCaMP3 up to at least 14 dpf. (A–C)** Epifluorescence images at 2 and 14 dpf of GCaMP3 expression in Tg[HuC:GCaMP3], Tg[HuC:Gal4VP16; 4xK + NnlsGC3] and Tg[HuC:Gal4VP16; 1xK + NnlsGC3]. Only Tg[HuC:Gal4VP16; 1xK + NnlsGC3] larvae exhibit robust GCaMP3 expression at 14 dpf.

Under epifluorescence, Tg[HuC:GCaMP3] larvae exhibited robust expression of GCaMP3 at 2 dpf that diminished significantly by 14 dpf (Figure 4A). Surprisingly, Tg[HuC:Gal4VP16; 4xK + NnlsGC3] larvae had very dim GCaMP3 expression at both 2 and 14 dpf (Figure 4B). However, our Tg[HuC:Gal4VP16; 1xK + NnlsGC3] larvae displayed robust expression of GCaMP3 at 2 dpf throughout the CNS that persisted to 14 dpf (Figure 4C). This suggests that the Kaloop mechanism using 1 UAS rather than 4 UAS repeats is preferable for driving brain-wide neuronal expression of GCaMP3 into later stages of larval development.

**Figure 5:**
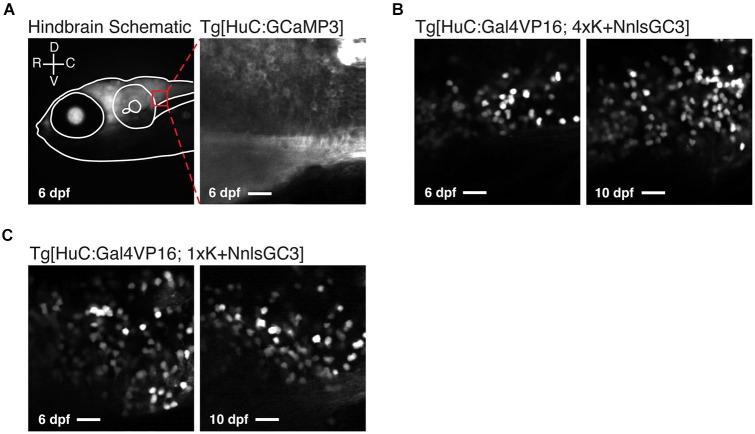
**Tg[HuC:Gal4VP16; 1xK + NnlsGC3] larvae exhibit mosaic nuclear expression of GCaMP3. (A)** Schematic of the 2PLSM imaging window in the caudal hindbrain (red box) overlaid on an epifluorescence image of a Tg[HuC:Gal4VP16; 1xK + NnlsGC3] taken at 6 dpf (left panel). Example 2PLSM fluorescence image of non-mosaic, dense cytoplasmic GCaMP3 expression in the caudal hindbrain of a Tg[HuC:GCaMP3] larva taken at 6 dpf (right panel). Scale bar represents 18 μm. **(B, C)** 2PLSM fluorescence images of the caudal hindbrain in Tg[HuC:Gal4VP16; 4xK + NnlsGC3] and Tg[HuC:Gal4VP16; 1xK + NnlsGC3] at 6 and 10 dpf. In **(B)** and **(C)**, highly mosaic expression of nuclear-localized GCaMP3 in Tg[HuC:Gal4VP16; 4xK + NnlsGC3] and Tg[HuC:Gal4VP16; 1xK + NnlsGC3] is evident. Scale bars represent 18 μm.

To assess the variegation of GCaMP3 labeling, we then looked at the caudal hindbrain under 2PLSM at 6 and 10 dpf. A schematic of a representative imaging window in the caudal hindbrain is shown in the left panel of Figure 5A. Imaging within such a window in Tg[HuC:GCaMP3] larvae typically revealed non-mosaic, dense expression of GCaMP3 (Figure 5A, right panel). Unexpectedly, both Tg[HuC:Gal4VP16; 4xK + NnlsGC3] and Tg[HuC:Gal4VP16; 1xK + NnlsGC3] larvae exhibited highly mosaic expression of GCaMP3 when examined with cellular resolution at both 6 and 10 dpf (Figures 5B,C). Note that the laser intensity was set for each strain to optimize visualization across days; the Tg[HuC:Gal4VP16; 4xK + NnlsGC3] strains required much greater laser power than the Tg[HuC:Gal4VP16; 1xK + NnlsGC3] strains, in accordance with the results from Figure 4.

We also looked at the subcellular localization of GCaMP3 across these strains. As expected, nuclear exclusion of GCaMP3 was clearly visible in Tg[HuC:GCaMP3] larvae under 2PLSM (Figure 5A, right panel). In Tg[HuC:Gal4VP16; 4xK + NnlsGC3] and Tg[HuC:Gal4VP16; 1xK + NnlsGC3] larvae, GCaMP3 was expressed in the nucleus of neurons, but also exhibited some expression in the cytoplasm (Figures 5B,C).

### Improved density of neuronal GCaMP3 expression with Tg[HuC:KalTA4]

**Figure 6:**
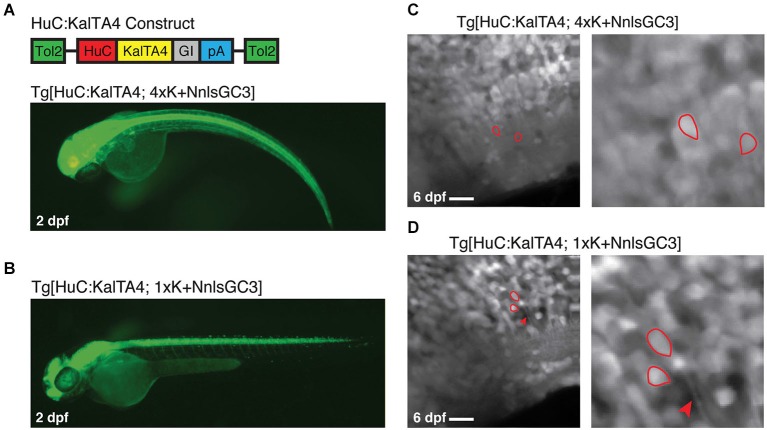
**Tg[HuC:KalTA4; 1xK + NnlsGC3] exhibits non-mosaic, dense expression of GCaMP3. (A, B)** Epifluorescence images of Tg[HuC:KalTA4; 4xK + NnlsGC3] and Tg[HuC:KalTA4; 1xK + NnlsGC3] at 2 dpf. **(C, D)** 2PLSM fluorescence images of the caudal hindbrain in Tg[HuC:KalTA4; 4xK + NnlsGC3] and Tg[HuC:KalTA4; 1xK + NnlsGC3] at 6 dpf. Example cells with both nuclear and cytoplasmic expression of GCaMP3 are outlined in red, and the red arrowhead in **(D)** points to a fluorescent neuronal process. Scale bars represent 18 μm.

To address the possibility that the presence of Gal4VP16 could be causing the observed mosaicism in our strains, we generated a Tg[HuC:KalTA4] driver line with ~panneuronal expression of KalTA4 instead of Gal4VP16. We then crossed this line to our Kaloop reporter strains to generate Tg[HuC:KalTA4; 4xK + NnlsGC3] and Tg[HuC:KalTA4; 1xK + NnlsGC3] larvae. Both double transgenic larval strains had robust expression of GCaMP3 at 2 dpf across the CNS (Figures 6A,B). However, the Tg[HuC:KalTA4; 4xK + NnlsGC3] larvae exhibited physical deformities whereas the Tg[HuC:KalTA4; 1xK + NnlsGC3] larvae did not. When examining these strains at cellular resolution in the caudal hindbrain with 2PLSM, densely packed GCaMP3 expression was observed (Figures 6C,D, left panels) in contrast to the mosaic expression seen in the lines expression Tg[HuC:Gal4VP16] (Figures 5B,C). Cells exhibiting low/no fluorescence were observed but were typically very few in number. This suggests that the existing ~panneuronal Gal4 driver line, Tg[HuC:Gal4VP16], causes highly mosaic expression when used with our NnlsGC3 Kaloop reporter strains. Our Tg[HuC:KalTA4] strain mitigates this mosaicism, most likely by reducing cell toxicity, to achieve a much higher density neuronal GCaMP3 expression.

As with larvae generated from crossing our Kaloop reporter strains with Tg[HuC:Gal4VP16], Tg[HuC:KalTA4; 4xK + NnlsGC3] and Tg[HuC:KalTA4; 1xK + NnlsGC3] larvae exhibited nuclear as well as some cytoplasmic GCaMP3 expression in neurons. Examples of cells with clear nuclear and cytoplasmic expression of GCaMP3 are shown outlined in red in the right panels of Figures 6C,D.

### Functional GCaMP3 signals in larvae generated with Tg[1xK + NnlsGC3]

We tested for GCaMP3 functionality in our strains using synchronous eye tracking and 2PLSM imaging in the caudal hindbrain of 6–8 dpf larval zebrafish. Neurons in this region comprise the oculomotor neural integrator, and persistently fire APs during fixations between saccades at a rate approximately linearly related to eye position (Pastor et al., 1994; Aksay et al., 2000; Miri et al., 2011b). Because intracellular Ca^2+^ concentration can be approximated as a filtered version of firing rate, Ca^2+^-dependent fluorescence fluctuations in eye position-encoding neurons should correlate with a similarly filtered version of eye position. Using a previously described linear regression-based approach (see Section Materials and Methods), we identified putative eye position-encoding neurons whose fluorescence fluctuations were correlated with appropriately filtered eye position.

**Figure 7:**
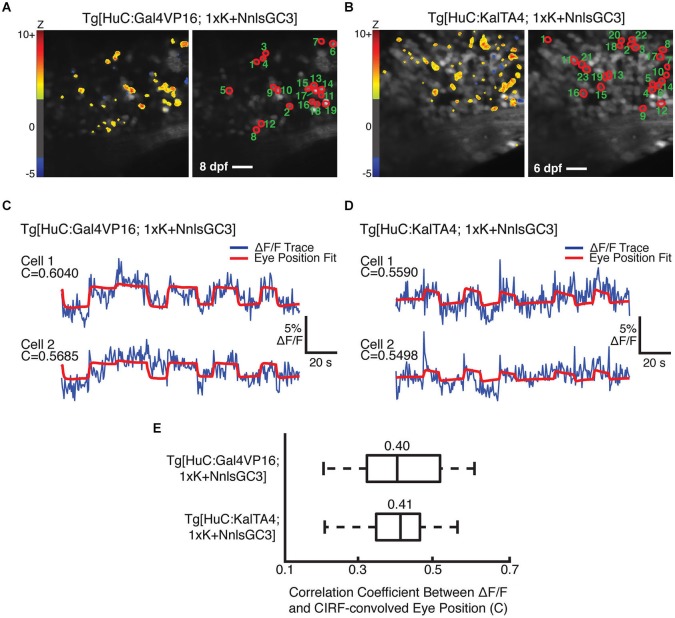
**Cells with behaviorally-correlated fluorescence fluctuations in Tg[HuC:Gal4VP16; 1xK + NnlsGC3] and Tg[HuC:KalTA4; 1xK + NnlsGC3]. (A, B)** Left panel: Pixel Z scores are mapped onto the mean images of 2PLSM fluorescence time series taken during spontaneous eye movements. Right panel: Cells whose 2PLSM fluorescence fluctuations are positively correlated with CIRF-convolved eye position are circled in red, and numbered in descending order of correlation coefficient, C. Scale bars represent 18 μm. **(C, D)** Overlaid 2PLSM fluorescence time series (blue) and the scaled CIRF-convolved eye position (red) for the first two cells. The CIRF-convolved eye position is scaled to minimize the squared difference from the fluorescence time series. **(E)** Summary of correlation coefficients for the identified cells in Tg[HuC:Gal4VP16; 1xK + NnlsGC3] and Tg[HuC:KalTA4; 1xK + NnlsGC3]. Vertical bars within the boxes represent the mean, box edges represent the 25th and 75th quartiles, and whiskers represent minima and maxima.

We were unable to identify behaviorally-correlated fluorescence fluctuations in Tg[HuC:GCaMP3] or Tg[HuC:Gal4VP16; 4xK + NnlsGC3] larvae, resulting from indicator expression that was too low in the former and perhaps too high in the latter. Imaging could not be performed in Tg[HuC:KalTA4; 4xK + NnlsGC3] larvae due to physical deformation, and thus these larvae could not be tested for GCaMP3 functionality. However, we were able to identify putative eye position-encoding cells in the mosaic Tg[HuC:Gal4VP16; 1xK + NnlsGC3] larvae and in the non-mosaic Tg[HuC:KalTA4; 1xK + NnlsGC3] larvae. Figures 7A,B show the outlines of identified cells (right panels) whose fluorescence fluctuations were significantly positively correlated with the CIRF-convolved eye position (Tg[HuC:Gal4VP16; 1xK + NnlsGC3]: *n* = 19 cells; Tg[HuC:KalTA4; 1xK + NnlsGC3]: *n* = 23 cells). The preponderance of cells exhibiting positive correlations (Figures 7A,B, left panels, warm colors) as opposed to negative correlations with eye position (blue colors; Tg[HuC:Gal4VP16; 1xK + NnlsGC3]: *n* = 1 cell; Tg[HuC:KalTA4; 1xK + NnlsGC3]: *n* = 3 cells) also suggests that these correlations are not due to motion artifacts (Miri et al., 2011b). Furthermore by visual inspection, the correlation between the cellular ΔF/F traces (Figures 7C,D, blue) and the CIRF-convolved eye position time series (red) is clearly evident. A summary of the correlation coefficients for the population of identified cells in Figures 7A,B are shown in Figure 7E (Tg[HuC:Gal4VP16; 1xK + NnlsGC3]: mean = 0.40; Tg[HuC:KalTA4; 1xK + NnlsGC3]: mean = 0.41). These results demonstrate that functional signals can be resolved from GCaMP3 using our transgenic strains. Thus our Tg[HuC:KalTA4] driver strain, or in general our transgenic strategy, could be used in the future to express improved Ca^2+^ indicators or optogenetic probes, enabling brain-wide optical imaging and/or perturbation into later developmental stages.

## Discussion

Calcium imaging is a powerful tool for measuring neuronal activity in large populations of neurons *in vivo*, yet its application in larval zebrafish has been hindered. The limited time window of ~panneuronal promoter activity has restricted the use of GECIs to early larval stages. Nuclear exclusion of GECIs reduces signal quality and resolution, locating much of the signal near the cytoplasmic membrane where it can be contaminated by signals from other cells. This is a particularly severe problem given the large nuclei and dense packing of somata in the developing zebrafish CNS. Here we report the generation of transgenic zebrafish strains enabling prolonged, brain-wide, nuclear-localized GCaMP3 expression using the previously described KalTA4-Kaloop system. The functionality of our GCaMP3 variants harboring an nls compared well to the non-localized original GCaMP3 in HEK cells *in vitro*. Our NnlsGC3 variant was able to reliably report APs in cultured neurons at kinetics ~2x as slow as non-nuclear GCaMP3. Furthermore our use of a novel Tg[HuC:KalTA4] strain markedly reduced the mosaic expression seen with an existing ~panneuronal driver strain when crossed with our new nuclear-localized GCaMP3 reporter strains. Finally we demonstrated that our new strains enable functional imaging in awake, behaving larvae by identifying putative eye position-encoding neurons in the caudal hindbrain. The strains we have developed are modular; they can be used in conjunction with different driver and reporter strains to drive the brain-wide expression of other probes or express nuclear-localized GCaMP3 in other cell populations later into development.

As many functional cell types within the larval zebrafish cannot be genetically targeted, ~panneuronal expression of calcium indicators is now commonly used to identify and study microcircuits in the larval zebrafish. However, existing approaches to ~panneuronal probe expression each have their own limitations. Documented panneuronal promoters such as HuC or α-1-tubulin (Goldman et al., 2001) are limited to early developmental time periods, whereas other constitutive promoters such as eno2 (Bai et al., 2007) fail to produce expression in a significant fraction of neurons (our unpublished observations). Furthermore, the Gal4^s1101t^ enhancer trap strain (Arrenberg et al., 2009), which allows ~panneuronal expression of Gal4VP16, causes mosaicism when coupled to reporter strains due to the effects of Gal4VP16. Therefore, our strategy for achieving robust neuronal expression throughout later stages of development could facilitate comprehensive, whole-brain scanning, which currently has only been achieved at early developmental stages in 5–7 dpf larvae (Ahrens et al., 2012, 2013; Freeman et al., 2014; Portugues et al., 2014; Vladimirov et al., 2014).

Larvae generated from crossing either Tg[1xK + NnlsGC3] or Tg[4xK + NnlsGC3] with Tg[HuC:Gal4VP16] or Tg[HuC:KalTA4] not only exhibited nuclear expression of GCaMP3, but also showed some fluorescence in the cytoplasm of neurons as well. The previously published nls we incorporated has been demonstrated to target a number of fluorescent proteins to the nuclei of zebrafish neurons (Kwan et al., 2007; Fujimoto et al., 2011); however, it is possible that when this nls is fused to a Ca^2+^ buffer, it does not localize to the nucleus as efficiently, perhaps at least in certain cellular contexts. The expression patterns we observed could also arise if the concentration of GCaMP3 overloaded the nuclear-targeting machinery, resulting in GCaMP3 lingering in the cytoplasm. Despite the additional cytoplasmic expression, nuclear inclusion of GCaMP3 in our strains still greatly increases the cross-sectional area from which fluorescence signals can be measured for a given larval zebrafish neuron, and also visibly reduces indicator concentration in the neuropil, which can potentially contaminate cellular signals measured from somata. Thus the indicator expression we have achieved should augment signal quality. Recently a nuclear-localized GCaMP variant was published using a histone H2B fusion (Freeman et al., 2014; Vladimirov et al., 2014). This nuclear targeting strategy appears to confine GCaMP expression almost entirely to the nucleus. However, a thorough characterization of this probe’s kinetics in response to APs has not been reported. Future studies utilizing an H2B fusion with our general transgenic strategy could prove useful for whole-brain imaging at later developmental stages. In addition, our nls-GCaMP3 probe can be used as a tool for relating nuclear Ca^2+^ signals to molecular or genetic changes *in vivo* or *in vitro*. For example, it is known that cytoplasmic and nuclear Ca^2+^ play distinct roles in controlling gene expression (Hardingham et al., 1997). The ability to measure nuclear Ca^2+^ signals throughout development can facilitate future studies examining how these gene expression changes are related to nuclear Ca^2+^ activity.

Surprisingly, we observed much more robust expression across the CNS in larvae generated from Tg[HuC:Gal4VP16] crossed with Tg[1xK + NnlsGC3] rather than crossed with Tg[4xK + NnlsGC3]. The mosaicism we observed with Tg[4xK + NnlsGC3] could be a result of transcriptional silencing resulting from methylation of UAS repeats (Goll et al., 2009). Another possibility is that high expression levels of GCaMP3 or KalTA4 stemming from the multiplicity of UAS repeats caused toxicity that selected for F1 larvae exhibiting highly mosaic expression. While Distel et al. (2009) did not observe mosaicism using Kaloop constructs containing four UAS repeats, they were not expressing a Ca^2+^ buffer and also were not driving expression across the entire brain, both of which could exacerbate toxicity. Although Gal4VP16 levels should not depend on UAS repeat number in the reporter cassette, Gal4VP16 may somehow permit or facilitate the mosaicism. In support of this, when we coupled Tg[4xK + NnlsGC3] with our novel Tg[HuC:KalTA4] strain instead of Tg[HuC:Gal4VP16], this greatly improved the expression level of GCaMP3.

While we were able to identify putative eye position-encoding cells using synchronous eye position tracking and 2PLSM imaging, the functional fluorescence fluctuations we identified were <5% in magnitude. One could argue that the small fluorescence signals may be an artifact of our predominantly nuclear Ca^2+^ signals; however, our *in vitro* studies found that the nuclear-localized GCaMP3 construct exhibited larger functional fluorescence signals than GCaMP3 limited to the cytoplasm. Furthermore, our previous studies *in vivo* using a bolus-loaded synthetic calcium indicator (which is expressed in both the cytoplasm and nucleus; Miri et al., 2011a,b) reported >50% functional fluorescence fluctuations, suggest that nuclear localization of the calcium sensor was not the cause of our small fluorescence signals. Instead, the signal quality we observed was perhaps due to limitations of GCaMP3 in larval zebrafish, which has been reported to produce small neuronal functional fluorescence signals *in vivo* (Del Bene et al., 2010). As small fluorescence signals can result in a poor signal-to-noise ratio and may mask the identification of behaviorally-correlated cells, larger functional Ca^2+^ signals are necessary to fully exploit this technique. To overcome this limitation, future Kaloop reporter strains generated with improved GECIs such as GCaMP6 (Chen et al., 2013) or GCaMP7 (Muto et al., 2013) should allow large-scale, chronic imaging of zebrafish into later larval stages when coupled to our Tg[HuC:KalTA4] strain.

## Author contributions

Christina K. Kim, Andrew Miri, David W. Tank, and Rebecca D. Burdine designed the experiments and interpreted results. Christina K. Kim and Andrew Miri performed cell culture and zebrafish experiments and analyzed data. Christina K. Kim, Andrew Miri, and Louis C. Leung generated transgenic zebrafish strains. Andre Berndt performed neuronal culture experiments. Christina K. Kim, Andrew Miri, and Rebecca D. Burdine wrote the paper.

## Conflict of interest statement

The authors declare that the research was conducted in the absence of any commercial or financial relationships that could be construed as a potential conflict of interest.

## References

[B1] Ahrens M. B. Li J. M. Orger M. B. Robson D. N. Schier A. F. Engert F.. (2012). Brain-wide neuronal dynamics during motor adaptation in zebrafish. Nature 485, 471–477. 10.1038/nature11057 22622571 PMC3618960

[B2] Ahrens M. B. Orger M. B. Robson D. N. Li J. M. Keller P. J. (2013). Whole-brain functional imaging at cellular resolution using light-sheet microscopy. Nat. Methods 10, 413–420. 10.1038/nmeth.2434 23524393

[B3] Aksay E. Baker R. Seung H. S. Tank D. W. (2000). Anatomy and discharge properties of pre-motor neurons in the goldfish medulla that have eye-position signals during fixations. J. Neurophysiol. 84, 1035–1049. 10938326 10.1152/jn.2000.84.2.1035

[B4] Appelbaum L. Wang G. Yokogawa T. Skariah G. M. Smith S. J. Mourrain P.. (2010). Circadian and homeostatic regulation of structural synaptic plasticity in hypocretin neurons. Neuron 68, 87–98. 10.1016/j.neuron.2010.09.006 20920793 PMC2969179

[B5] Arrenberg A. B. Del Bene F. Baier H. (2009). Optical control of zebrafish behavior with halorhodopsin. Proc. Natl. Acad. Sci. U S A 106, 17968–17973. 10.1073/pnas.0906252106 19805086 PMC2764931

[B6] Bai Q. Garver J. A. Hukriede N. A. Burton E. A. (2007). Generation of a transgenic zebrafish model of Tauopathy using a novel promoter element derived from the zebrafish *eno2* gene. Nucleic Acids Res. 35, 6501–6516. 10.1093/nar/gkm608 17897967 PMC2095798

[B7] Baron U. Gossen M. Bujard H. (1997). Tetracyclin-controlled transcription in eukaryotes: novel transactivators with graded transactivation potential. Nucleic Acids Res. 25, 2723–2729. 10.1093/nar/25.14.2723 9207017 PMC146828

[B8] Beck J. C. Gilland E. Tank D. W. Baker R. (2004). Quantifying the ontogeny of optokinetic and vestibuloocular behaviors in zebrafish, medaka and golfish. J. Neurophysiol. 92, 3546–3561. 10.1152/jn.00311.2004 15269231

[B9] Bengtson C. P. Freita H. E. Weislogel J. Bading H. (2010). Nuclear calcium sensors reveal the repetition of trains of synaptic stimuli boosts nuclear calcium signaling in CA1 pyramidal neurons. Biophysiol. J. 99, 4066–4077. 10.1016/j.bpj.2010.10.044 21156150 PMC3000507

[B10] Berndt A. Lee S. Y. Ramakrishnan C. Deisseroth K. (2014). Structure-guided transformation of Channelrhodopsin into a light-activated chloride channel. Science 344, 420–424. 10.1126/science.1252367 24763591 PMC4096039

[B11] Chen T.-W. Wardill T. J. Sun Y. Pulver S. R. Renninger S. L. Baohan A.. (2013). Ultrasensitive fluorescent proteins for imaging neuronal activity. Nature 499, 295–300. 10.1038/nature12354 23868258 PMC3777791

[B12] Del Bene F. Wyart C. Robles E. Tran A. Looger L. Scott E. K.. (2010). Filtering of visual information in the tectum by an identified neural circuit. Science 330, 669–673. 10.1126/science.1192949 21030657 PMC3243732

[B13] Distel M. Wullimann M. F. Köster R. W. (2009). Optimized Gal4 genetics for permanent gene expression mapping in zebrafish. Proc. Natl. Acad. Sci. U S A 106, 13365–13370. 10.1073/pnas.0903060106 19628697 PMC2726396

[B14] Freeman J. Vladimirov N. Kawashima T. Mu Y. Sofroniew N. J. Bennett D. V.. (2014). Mapping brain activity at scale with cluster computing. Nat. Methods 11, 941–950. 10.1038/nmeth.3041 25068736

[B15] Fujimoto E. Gaynes B. Brimley C. J. Chien C. B. Bonkowsky J. J. L. (2011). Gal80 intersectional regulation of cell-type specific expression in vertebrates. Dev. Dyn. 240, 2324–2334. 10.1002/dvdy.22734 21905164 PMC3178006

[B16] Goldman D. Hankin M. Li Z. Dai X. Ding J. (2001). Transgenic zebrafish for studying nervous system development and regeneration. Transgenic Res. 10, 21–33. 10.1023/A:1008998832552 11252380

[B17] Goll M. G. Anderson R. Stainier D. Y. R. Spradling A. C. Halpern M. E. (2009). Transcriptional silencing and reactivation in transgenic zebrafish. Genetics 182, 747–755. 10.1534/genetics.109.102079 19433629 PMC2710156

[B18] Hardingham G. E. Chawla S. Johnson C. M. Bading H. (1997). Distinct functions of nuclear and cytoplasmic calcium in control of gene expression. Nature 385, 260–265. 10.1038/385260a0 9000075

[B19] Higashijima S. Masino M. A. Mandel G. Fetcho J. R. (2003). Imaging neuronal activity during zebrafish behavior with a genetically encoded calcium indicator. J. Neurophysiol. 90, 3986–3997. 10.1152/jn.00576.2003 12930818

[B20] Hocking J. C. Distel M. Köster R. W. (2012). Studying cellular and subcellular dynamics in the developing zebrafish nervous system. Exp. Neurol. 242, 1–10. 10.1016/j.expneurol.2012.03.009 22465266

[B21] Jetti S. K. Vendrell-Llopis N. Yaksi E. (2014). Spontaneous activity governs olfactory representations in spatially organized habenular microcircuits. Curr. Biol. 24, 434–439. 10.1016/j.cub.2014.01.015 24508164

[B22] Kawakami K. (2005). Transposon tools and methods in zebrafish. Dev. Dynam. 234, 244–254. 10.1002/dvdy.20516 16110506

[B23] Kimura Y. Satou C. Higashijima S. (2008). V2a and V2b neurons are generated by the final divisions of pair-producing progenitors in the zebrafish spinal cord. Development 135, 3001–3005. 10.1242/dev.024802 18684740

[B24] Kinkhabwala A. Riley M. Koyama M. Monen J. Satou C. Kimura Y.. (2011). A structural and functional ground plan for neurons in the hindbrain of zebrafish. Proc. Natl. Acad. Sci. U S A 108, 1164–1169. 10.1073/pnas.1012185108 21199947 PMC3024665

[B25] Köster R. W. Fraser S. E. (2001). Tracing transgene expression in living zebrafish embryos. Dev. Biol. 233, 329–346. 10.1006/dbio.2001.0242 11336499

[B26] Kwan K. M. Fujimoto E. Grabher C. Mangum B. D. Hardy M. E. Campbell D. S.. (2007). The Tol2kit: a multisite gateway-based construction kit for Tol2 transposon transgenesis constructs. Dev. Dyn. 236, 3088–3099. 10.1002/dvdy.21343 17937395

[B27] Lister J. A. Robertson C. P. Lepage T. Johnson S. L. Raible D. W. (1999). Nacre encodes a zebrafish microphthalmia-related protein that regulates neural-crest-derived pigment cell fate. Development 126, 3757–3767. 10433906 10.1242/dev.126.17.3757

[B28] Liu C. Hermann T. E. (1978). Characterization of ionomycin as a calcium ionophore. J. Biol. Chem. 253, 5892–5894. 28319

[B29] Miri A. Daie K. Arrenberg A. B. Baier H. Aksay E. Tank D. W. (2011a). Spatial gradients of firing rate persistence in a neural integrator circuit. Nat. Neurosci. 14, 1150–1159. 10.1038/nn.2888 21857656 PMC3624014

[B30] Miri A. Daie K. Burdine R. D. Aksay E. Tank D. W. (2011b). Regression-based identification of behavior-encoding neurons during large-scale optical imaging of neural activity at cellular resolution. J. Neurophysiol. 105, 964–980. 10.1152/jn.00702.2010 21084686 PMC3059183

[B31] Muto A. Ohkura M. Abe G. Nakai J. Kawakami K. (2013). Real-time visualization of neuronal activity during perception. Curr. Biol. 23, 307–311. 10.1016/j.cub.2012.12.040 23375894

[B32] Naumann E. A. Kampff A. R. Prober D. A. Schier A. F. Engert F. (2010). Monitoring neural activity with bioluminescence during natural behavior. Nat. Neurosci. 13, 513–520. 10.1038/nn.2518 20305645 PMC2846983

[B33] Park H. C. Kim C. H. Bae Y. K. Yeo S. Y. Kim S. H. Hong S. K.. (2000). Analysis of upstream elements in the HuC promoter leads to the establishment of transgenic zebrafish with fluorescent neurons. Dev. Biol. 227, 279–293. 10.1006/dbio.2000.9898 11071755

[B34] Pastor A. M. De la Cruz R. R. Baker R. (1994). Eye position and eye velocity integrators reside in separate brainstem nuclei. Proc. Natl. Acad. Sci. U S A 91, 807–811. 10.1073/pnas.91.2.807 8290604 PMC43038

[B35] Pologruto T. A. Sabatini B. L. Svoboda K. (2003). ScanImage: flexible software for operating laser scanning microscopes. Biomed. Eng. Online 2:13. 10.1186/1475-925X-2-13 12801419 PMC161784

[B36] Portugues R. Feierstein C. E. Engert F. Orger M. B. (2014). Whole-brain activity maps reveal stereotyped, distributed networks for visuomotor behavior. Neuron 81, 1328–1343. 10.1016/j.neuron.2014.01.019 24656252 PMC4448760

[B37] Provost E. Rhee J. Leach S. D. (2007). Viral 2A peptides allow expression of multiple proteins from a single ORF in transgenic zebrafish embryos. Genesis 45, 625–629. 10.1002/dvg.20338 17941043

[B38] Sato T. Takahoko M. Okamoto H. (2006). HuC:Kaede, a useful tool to label neural morphologies in networks *in vivo*. Genesis 44, 136–142. 10.1002/gene.20196 16496337

[B39] Schrödel T. Prevedel R. Aumayr K. Zimmer M. Vaziri A. (2013). Brain-wide 3D imaging of neuronal activity in *Caenorhabditis elegans* with sculpted light. Nat. Methods 10, 1013–1020. 10.1038/nmeth.2637 24013820

[B40] Scott E. K. Mason L. Arrenberg A. B. Ziv L. Gosse N. J. Xiao T.. (2007). Targeting neural circuitry in zebrafish using GAL4 enhancer trapping. Nat. Methods 4, 323–326. 10.1038/nmeth1033 17369834

[B41] Stosiek C. Garaschuk O. Holthoff K. Konnerth A. (2003). In vivo two-photon calcium imaging of neuronal networks. Proc. Natl. Acad. Sci. U S A 100, 7319–7324. 10.1073/pnas.1232232100 12777621 PMC165873

[B42] Thomas P. Smart T. G. (2005). HEK293 cell line: a vehicle for the expression of recombinant proteins. J. Pharmacol. Toxicol. Methods 51, 187–200. 10.1016/j.vascn.2004.08.014 15862464

[B43] Tian L. Hires S. A. Mao T. Huber D. Chiappe M. E. Chalasani S. H.. (2009). Imaging neural activity in worms, flies and mice with improved GCaMP calcium indicators. Nat. Methods 6, 875–881. 10.1038/nmeth.1398 19898485 PMC2858873

[B44] Vladimirov N. Mu Y. Kawashima T. Bennett D. V. Yang C. Looger L. L.. (2014). Light-sheet functional imaging in fictively behaving zebrafish. Nat. Methods 11, 883–884. 10.1038/nmeth.3040 25068735

[B45] Walker A. S. Burrone J. Meyer M. P. (2013). Functional imaging in the zebrafish retinotectal system using RGECO. Front. Neural Circuits 7:34. 10.3389/fncir.2013.00034 23508811 PMC3589694

[B46] Warp E. Agarwal G. Wyart C. Friedmann D. Oldfield C. S. Conner A.. (2012). Emergence of patterned activity in the developing zebrafish spinal cord. Curr. Biol. 22, 93–102. 10.1016/j.cub.2011.12.002 22197243 PMC3267884

